# Evaluating land suitability and water availability for surface irrigation in the Abbay basin of Ethiopia

**DOI:** 10.1098/rsos.220674

**Published:** 2022-12-14

**Authors:** Yilkal A. Kassie, Abdu Y. Yimam, Tewodros T. Assefa, Sisay A. Belay

**Affiliations:** ^1^ School of Civil and Water Resource Engineering, Woldia University, Woldia, Amhara 400, Ethiopia; ^2^ Faculty of Civil and Water Resource Engineering, Bahir Dar Institute of Technology, Bahir Dar University, Bahir Dar 26, Ethiopia

**Keywords:** land suitability, surface and groundwater potential, analytical hierarchy process, surface irrigation, Abbay basin

## Abstract

This study was conducted in the Abbay basin of Ethiopia to evaluate land suitability for irrigation considering both surface and groundwater sources using the analytic hierarchy process. Multiple factors which affect irrigated agriculture productivity were considered, and an 85% threshold was applied to identify irrigable land. The suitability result was validated using ground truth data from existing irrigation projects for surface water sources and depth to groundwater data for groundwater sources. The low flow potential of rivers, which is dependable for surface irrigation, was evaluated against suitable land considering the most dominant crops. The result showed that nearly 10% of the basin area (19 192 km^2^) and 5.3% of the basin (10 364 km^2^) were found suitable for surface irrigation from rivers and groundwater, respectively. South Gojam was found to be the most suitable sub-basin (approx. 3880 km^2^) for surface irrigation, whereas Muger was found to be the most suitable sub-basin (approx. 2105 km^2^) for surface irrigation from rivers and groundwater, respectively. Depth to groundwater was shallow for Muger as compared with other sub-basins. The validation result depicted more than 83% and 73% overlap for surface and groundwater sources, respectively. Land suitability and water availability assessment result in the Abbay basin shows a promising result for surface irrigation developments.

## 1. Introduction

Agriculture in Ethiopia contributes 43% of the national gross domestic product (GDP) [1,2]. The crop production system is mainly on rain-fed agriculture with limited irrigation practice [3,4]. Rainfall variability frequently hampers the rain-fed production system in the nation [5,6]. Consequently, food insecurity and frequent drought caused by the unreliable rainfall distribution are common, which affect the livelihoods of the rural community [7,8], depicting the need to expand irrigated agriculture for sustainable food supply in the nation [9,10]. Adequate suitable land resources and water availability are key to transforming the rain-fed agricultural system into irrigated agriculture [11,12].

Irrigated agriculture is critical to reducing the adverse effect of rainfall variability and improving crop production [13,14]. However, understanding the potential of water resources (surface water and groundwater source) is essential for the proper planning and development of irrigation projects [15,16]. Surface water has been used as the main source of irrigation while the use of groundwater is limited to domestic use [17–19]. Groundwater is more reliable for irrigation than surface water, due to its slow reaction to climate change and contaminants [20]. Besides, a recent study [19] showed that about 8% of the irrigable land in Ethiopia could be addressed using only shallow groundwater. Even though groundwater remains a reliable resource, the lack of technology and information on the potential of the available resource hampers its use for irrigation [20,21].

Water resource planning for surface irrigation requires information about the land suitability, water availability and water requirements of crops in irrigable areas in time and space [22,23]. Evaluating the potential of water resources and areas suitable for irrigation purposes helps to increase water and crop productivity and cost-effective use of land resources [24,25]. On the other hand, identifying suitable land for surface irrigation requires the evaluation of multiple factors that affect irrigated agriculture using multi-criteria evaluation (MCE) in a geographic information system (GIS) environment.

The GIS-based MCE technique has been used in several water resource studies such as hydropower potential site selection [26,27], erosion hotspot identification [28–31], irrigation suitable site assessment [32,33] and wastewater disposal site selection [34–37]. Similarly, there have been many studies conducted on the assessment of irrigable land potential [38–41] in the Ethiopian highlands. In the Shaya river sub-basin of the Oromiya region, around 47.3% of the land was found suitable for irrigation [22]. Similarly, 9% of land suitable for irrigation was estimated in the Gilo sub-basin of Gambella [39]. Birhanu *et al*. [41] reported significant suitable land for irrigation in the Dirma river basin (68.3%). In the Rib and Gumara watershed of North Ethiopia, around 93% of the land was estimated suitable for surface irrigation developments [38]. Assefa *et al*. [20] evaluated potentially irrigable areas for home gardens and found about 1.2% of the irrigable land of the Lake Tana basin could be addressed from rivers and up to 2.4% of the irrigable land from groundwater operated under conventional irrigation techniques. Worqlul *et al*. [42] found around 20% of the Lake Tana basin is suitable for surface irrigation. Our study focused on the Abbay basin, which has been designated as one of the growth corridors for economic development by the government of Ethiopia to end poverty [43]. The basin covers 20% of the nation's land area, 50% of the country's surface water resources, 25% of the nation's population and over 40% of the nation's agricultural product [44]. In addition, a significant amount of run-off from the highlands to the downstream countries, especially to Sudan and Egypt has been contributed from the Abbay basin [45] which needs investigation on its potential for irrigation.

There is a little study conducted on the investigation of surface irrigation potential from surface water sources in the Abbay basin by Yimere & Assefa [46] using GIS-MCE techniques and Yalew *et al*. [44] using the Mike Hydro model considering existing irrigation projects. In this study, we integrated assessment of land suitability and water availability for irrigation considering multiple factors which are lacking currently in the basin. Besides, the low flow potential of the major rivers in the Abbay basin for surface irrigation has been addressed. The results from this study would help local decision-makers and stakeholders by providing evidence of the potential expansion of small-scale irrigated agriculture to help improve both water and land productivity.

## 2. Study area

**Figure 1.**
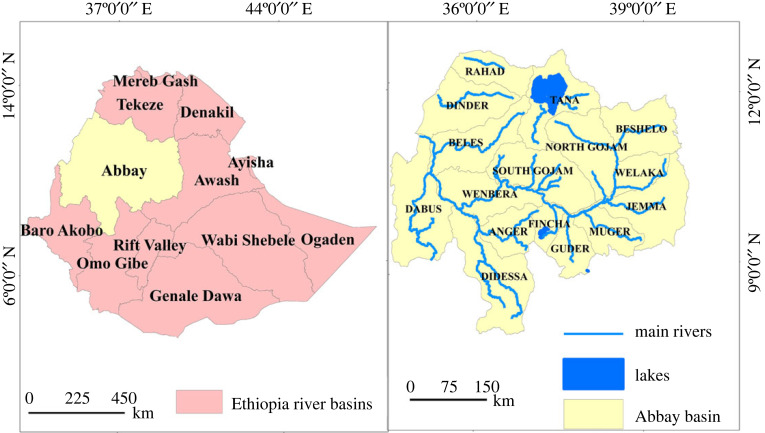
Map of the study area (Abbay basin of Ethiopia).

Abbay basin, often called Blue Nile basin, (figure 1) is located in the Northwest part of Ethiopia from latitude 70°45′ to 120°46′ N and from longitude 340°06′ to 400°00′ E. The basin extends about 400 km from north to south and about 550 km from east to west having a total surface area coverage of 199 812 km^2^. It shares a boundary with the Tekeze basin to the north, the Awash basin to the east and the Baro-Akobo basin to the southwest. The country's largest freshwater lake and the source of the Abbay (Blue Nile) River, Lake Tana, are located to the north of the basin. Abbay basin is the second largest river basin in Ethiopia next to the Wabishebele basin. It has the largest share in terms of annual run-off potential (54.8 billion m^3^). The basin accounts for more than 28% of Ethiopia's total population, 45% of the surface water resource, 40% of the agricultural product and most of the hydropower and irrigation potential [47]. The basin contributes over 40% of the nation's agricultural product [44]. The basin has been chosen as a growth corridor to achieve socio-economic development because of its huge potential [48].

The dominant soil texture of the entire basin is heavy clay [49] and the major soil types are Luvisols, Vertisols, Nitisols, Leptosols, Gelysolsl and Fluvisols. The rainfall season runs mostly from June to September with a range of annual rainfall variation from 779 to 2457 mm, observed from the recordings of 10 years of meteorological data (2008–2018). The average air temperature also ranges from 16 to 25°C across the basin. Its altitude ranges from 483 (western part of the basin) to 4266 m.a.s.l. (eastern part of the basin), done during digital elevation model (DEM) preparation. Residents practised irrigation mostly by traditionally diverting rivers and manual lifting of groundwater to supplement the rain-fed production system both from groundwater and surface water sources [50].

Rope and washer systems, pulley systems and bucket systems have been practised in some parts of the sub-basins to extract groundwater and used for drinking and home garden vegetable production. The dominant vegetables cultivated in the region are onion, carrot, potato, sugarcane, garlic, tomato, pepper and beetroot [20,51]. Similarly, the dominant cereals grown across the basin are sorghum, wheat and maize [52]. Irrigation from surface water sources has been implemented to some extent through small- to large-scale irrigation projects constructed by the government [53]. Among the large-scale irrigation projects, Fincha, Ribb, Koga, Gilgel Abbay, Anger, Tiss Abbay, Jemma, Beles and Dedissa irrigation projects were constructed to maximize productivity. The maximum irrigable land from these large irrigation projects was around 80 000 ha. In addition, there are small-scale irrigation projects, weirs, constructed by the regional government.

## 3. Material and methods

**Figure 2.**
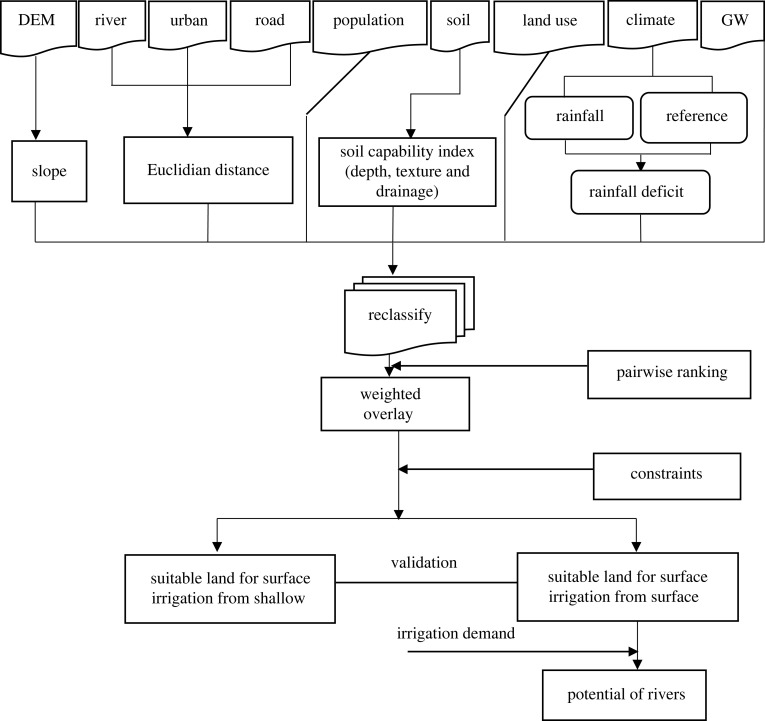
Framework to identify suitable land for irrigation from surface and groundwater (GW).

First, suitable land for irrigation was identified in the Abbay basin from both surface and shallow groundwater sources using the multi-criteria evaluation (MCE) technique (figure 2). Shallow groundwater for this study is defined as any subsurface water accumulated in a well depth of less than 25 m [19]. Soil capability index (SCI), land use/land cover, slope, rainfall deficit, proximity to urban centres and road networks and population density were considered as major factors commonly used for suitability evaluation, both from surface and groundwater sources [19,42]. In addition, proximity to the river network was considered for land suitability evaluation from the surface water, whereas depth to groundwater and salinity were considered for land suitability evaluation from groundwater sources. Several spatial and temporal data (table 1) were used in this study to achieve the research objectives. The factors were reclassified based on their suitability ranges (table 2) for surface irrigation. The pairwise ranking technique was used to develop weights for the factors comparing one-to-one (comparison matrix) separately for surface water source [40,42] and groundwater source [19,20,54]. The weighted overlay was used to identify potentially irrigable land from the surface and groundwater sources. A constraint map was developed from water bodies, wetlands, urban areas and protected areas. Potentially irrigable land was multiplied with a constraint map which is 0 (constraint) and 1 (can be used for irrigation) to limit irrigation in the constraint areas. An 85% threshold value was used based on Worqlul *et al*. [19] and Assefa *et al*. [20] to identify suitable areas for irrigation. In the second stage, the land suitability evaluation result was validated using information from shallow groundwater wells (depth) and currently irrigated lands from rivers. In the third stage, a case study was conducted to evaluate the potential of main rivers to address suitable land for irrigation considering the irrigation requirements of dominant crops grown in the basin, and the low flow (90 percentile flow) of rivers based on Yimam *et al*. [55].

**Table 1.** Spatial and temporal data used in this study and their sources.

data type	source	spatial resolution (m)
digital elevation model (DEM)	United States Geological Survey (USGS)	30
land use land/cover	Abbay Basin Authority	30
soil	Africa Soil Information Services (AfSIS)	250
population density	Global Gridded Population Database	1000
groundwater depth and salinity	British Geological Survey (BGS), for validation	5000
existing groundwater depth for validation	Amhara National Regional State Water Resources Bureau	—
road network, the network of towns and the river network	Abbay Basin Authority	—
climate	Ethiopian National Meteorological Agency	—
stream discharge	Abbay Basin Authority	—
irrigated land in Abbay basin	Abbay Basin Authority	—

**Table 2.** A general framework of land suitability classification for irrigation [20].

class	name	land description
S1	highly suitable	land without significant limit; this land is the best possible and does not reduce productivity or required increased inputs
S2	moderately suitable	land that is suitable but has a limitation that either reduces productivity or requires an increase of inputs to sustain productivity compared with S1
S3	marginally suitable	land with limitations so severe that benefits are reduced and/or the input required to sustain production needs to be increased so that this cost is only marginally justified
S4 (N1)	currently not suitable	land having limitations that may be surmountable in time but which cannot be corrected with existing knowledge at a currently acceptable cost
S5 (N2)	permanently not suitable	land having limitations that appear as severe as to preclude any possibilities of successful sustained use of the land of a given land use

### 3.1. Slope

The slope of land has a major impact on irrigation suitability in terms of land preparation and irrigation operation and efficiency [56]. A 30 m DEM was used to drive the slope map of the study area for irrigation suitability analysis. The slopes were reclassified based on their suitability for surface irrigation according to [42] into five classes; highly suitable (0–2%), moderately suitable (2–8%), marginally suitable (8–12%), less suitable (12–30%) and currently not suitable (greater than 30%).

### 3.2. Soil capability index

The suitability of soil for sustained production of cultivated crops which depends on soil depth, soil texture and soil drainage was expressed using the SCI [57], which describes the ability of soil for irrigation practices [20]. The data from AfSIS are available with 250 m resolution across depth; 0–5 cm, 5–15 cm, 15–30 cm, 30–60 cm, 60–100 cm and 100–200 cm. The depth-wise soil physical properties were aggregated by weighted average techniques and classified according to the United States Department of Agriculture (USDA) soil classification method to get the texture, depth and drainage of the entire Abbay basin soils considering the maximum root depth of crops grown in the area (100 cm). The suitability of the soil for irrigation was determined by the SCI using equation (3.1) [58].

3.1 $$\mathrm{SCI}=\;A\;\times \;\left(\frac{B}{100}\right)\times \;\left(\frac{C}{100}\right)\;,$$

where *A*, *B* and *C* are soil texture rating, soil depth rating and soil drainage class rating, respectively. The SCI was reclassified according to Teka *et al*. [58], as highly suitable (greater than 80%), moderately suitable (60–80%), marginally suitable (45–60%), less suitable (30–40%) and currently not suitable (less than 30%).

#### 3.2.1. Soil texture rating

**Figure 3.**
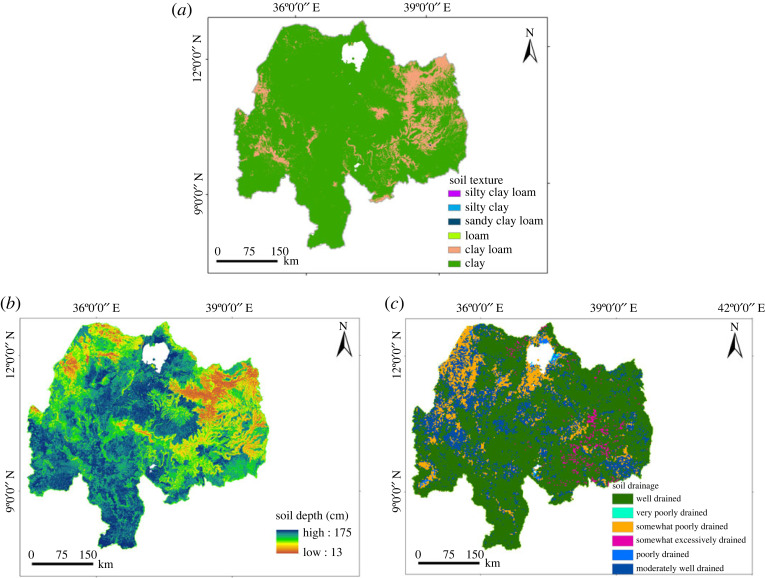
Soil capability indexing factors in the Abbay basin; (*a*) soil texture, (*b*) soil depth (cm) and (*c*) soil drainage.

The textural class for the Abbay basin was clay, clay loam, loam, sandy clay loam, silty clay and silty clay loam (figure 3*a*). The soil water-holding capacity was taken as the most important parameter for irrigation [59] and used for suitability classification in this study. According to FAO [59], soil textures were classified into four based on suitability for irrigation practice considering water-holding capacity as a suitability indicator. These are highly suitable (silt, silt loam and silty clay loam), moderately suitable (silty clay and clay), marginally suitable (sandy clay loam) and not suitable (sand).

#### 3.2.2. Soil depth rating

FAO [60] standard guidelines for evaluation of surface irrigation were used for soil depth (figure 3*b*) suitability classification considering more water and nutrient be provided for plants in a deeper soil than in shallow soil depth. Consequently, the soil depth was classified into four suitability classes: highly suitable (greater than 100 cm), moderately suitable (80–100 cm), marginally suitable (50–80 cm) and not suitable (less than 50 cm).

### 3.3. Soil drainage rating

Soil drainage is important for good aeration in plant roots and better growth and productivity of irrigated crops. The soil drainage (figure 3*c*) for the Abbay basin has six classes; well-drained, moderately well-drained, somewhat excessively drained, poorly drained, somewhat poorly drained and very poorly drained soils. The soil drainage classes were reclassified into four suitability classes, based on FAO [60], which are a function of soil depth. Thus, the suitability classes are well-drained to moderately drained soil (depth greater than 100 cm), imperfectly drained soil (depth 80–100 cm), poorly drained soil (depth 50–80 cm) and very poorly drained soil (depth less than 50 cm). The order of suitability classes was from highly suitable (the former) to currently not suitable (the latter).

### 3.4. Land use and land cover

**Figure 4.**
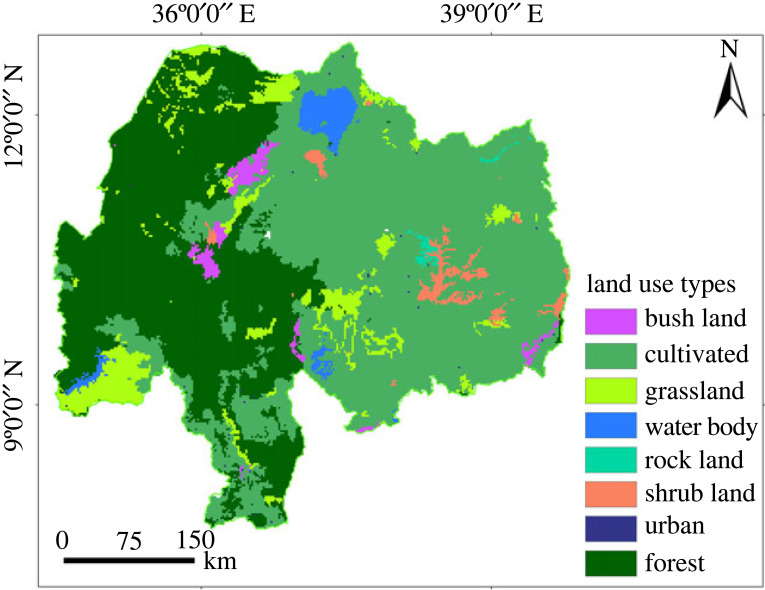
Land use and land cover of Abbay basin.

The land-use pattern of a watershed has a strong impact on water quality and water resource availability for irrigation practice and also for irrigation operation [61]. The Abbay basin land-use types (figure 4) were reclassified based on FAO [60] guidelines ranging from highly suitable to permanently not suitable based on the cost of land preparation for agriculture and constraints (urban area, water body, wetlands). Accordingly, agricultural land is classified as highly suitable, followed by grassland, which requires land clearing and levelling as moderately suitable land. Shrub and bare land require a higher initial investment and are classified as marginally suitable land. Rock land, woodland, bamboo, forest and plantations were currently not suitable classes, whereas, water, urban and wetland were permanently not suitable classes.

### 3.5. Population density and proximity to urban, road and rivers

Market outlets having easy access to roads and proximity to the urban centres with densely populated areas are regarded as highly suitable for irrigation to access agricultural inputs and sell products. In addition, distance from rivers is influential for surface irrigation from rivers. Geometric interval ranging techniques were used to reclassify the suitable land for irrigation due to the high variability of the population density data based on Huan *et al*. [62]. Proximity to the road network, urban centres and river networks was computed using Euclidean distance and reclassified using the equal interval ranging technique based on Teshome & Halefom [63]. Reclassification was made into eight classes for each factor based on Worqlul *et al*. [19].

### 3.6. Rainfall deficit

**Figure 5.**
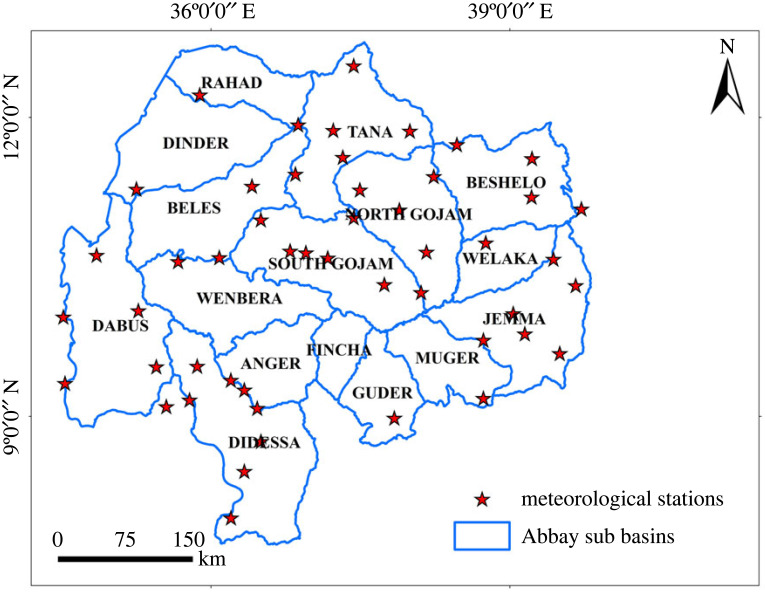
Meteorological stations in the Abbay basin.

Rainfall deficit is one of the harmful effects of climatic shocks which affect agricultural productivity and guide the use of irrigation practices to improve productivity [64]. A positive rainfall deficit value indicates that the rainfall is higher than evapotranspiration and a sufficient amount of water is available from rainfall, indicating irrigation supplement is not needed. Whereas, the negative rainfall deficit values indicate that evapotranspiration is higher than rainfall, indicating the need for additional irrigation water for crop production, which is the case for this study. Ten-year monthly rainfall, temperature, wind speed, sunshine hour and relative humidity of 58 meteorological stations were collected from the Ethiopian National Meteorological Agency (ENMA) from the years 2008 to 2018 (figure 5). The monthly rainfall was aggregated on an annual basis for each station. The potential evapotranspiration was computed using the Penman–Monteith method [65] as shown in equation (3.2) for each station and aggregated into annual values for each station. The inverse distance weighting (IDW) technique [66] was applied to map both rainfall and evapotranspiration on a basin scale, and rainfall deficits were computed by deducting evapotranspiration from rainfall. The arithmetic averaging technique and normal mean ratio method [67] were used to fill the missing data. The rainfall deficit was reclassified into five classes based on the equal interval ranging technique.

3.2 $${\mathrm{ET}}_{o}=\frac{0.408\Delta (R_{n}\;-\;G)\;+\;\gamma \frac{900}{T\;+\;273}\;u_{2}(e_{s}\;-\;e_{a})}{\Delta \;+\;\gamma (1\;+\;0.34u_{2})}.$$

where ET_o_, *R_n_*, *G*, *T*, *u*_2_, *e_s_*, *e_a_*, Δ and *γ* are reference evapotranspiration (mm d^−1^), net radiation at the crop surface (MJ m^−2^ d^−1^), soil heat flux density (MJ m^−2^ d^−1^), air temperature (°C), wind speed at 2 m height (m s^−1^), saturation vapour pressure (KPa), actual vapour pressure (KPa), slope vapour pressure curve (KPa °C^−1^) and psychometric constant (KPa °C^−1^), respectively.

### 3.7. Groundwater depth and salinity

**Figure 6.**
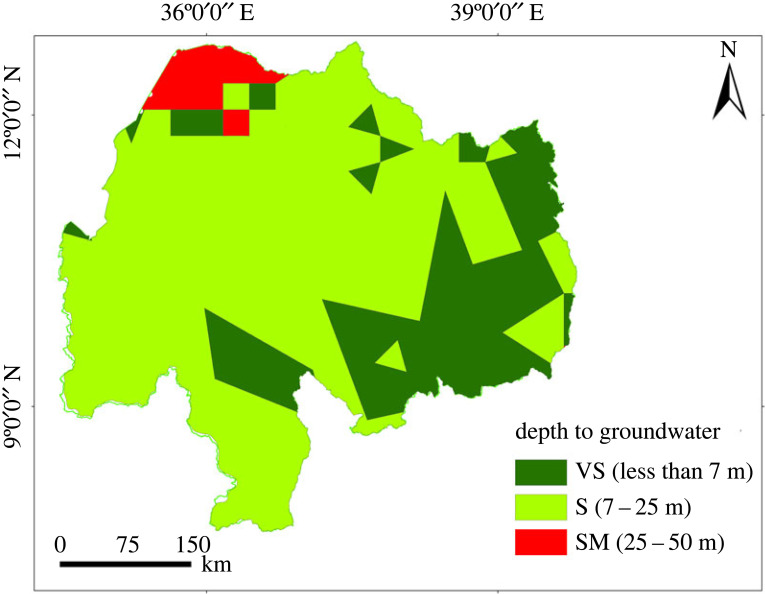
Depth to groundwater in the Abbay basin.

Groundwater is an important source of water for irrigation where surface water is relatively scarce, depending on the salt concentration [68], in which low-salinity areas have greater productivity from irrigation [69]. Depth to groundwater was used to evaluate the land suitability for irrigation using a groundwater source (figure 6). The depth of groundwater was reclassified into suitability classes based on the effort needed to extract water [20], and only shallow groundwater was considered for this study. In addition, the concentration of salt in the groundwater was also obtained from BGS, and its suitability for irrigation was judged based on the concentration of total dissolved solids (TDSs) according to Singh & Singh [70].

### 3.8. Constraint to irrigation

A constraint serves to limit the alternatives under consideration. In many cases, constraints are areas excluded from the consideration that should be coded with a zero, and those open for consideration should be also coded with a one. In this study, permanently unsuitable land (constraint) such as water bodies, wetlands, major paved roads, urban/built-up areas and protected areas were considered as constraints for physical land suitability for irrigation. A constraint map with a value of 0 and 1 was used to exclude the non-suitable areas from the preliminary suitable land. A constraint map (map with 0 and 1 values) was developed and multiplied with the developed suitability map to avoid limitations (water body, wetland, urban areas, forest and protected areas) that influence surface irrigation practice.

### 3.9. The pairwise ranking technique

The pairwise ranking technique [71] was used to assign a weight to the factors comparing them on a one-to-one basis using a scale from 1 to 9 (table 3). The highest value (9) refers to absolute importance, whereas, the lowest value (1) refers to equal importance and is reciprocated in the transpose position to indicate insignificant. The pairwise comparison matrix was adopted from [19,20,42,54]. The analytical hierarchical process (AHP) [73] was applied to compute the final suitability mapping for surface irrigation. The overall weights of the factors were distributed to individual factor suitability classes using the equal interval ranging technique.

**Table 3.** Pair wise comparison matrix scaling [72].

intensity of importance	definition	explanation
1	equal importance	two factors contribute equally to the objective
3	somewhat more important	experience and judgement are slightly favourable one over the other
5	much more important	experience and judgement strongly favour one over the other
7	very much more important	experience and judgement strongly favour one over the other; its importance is demonstrated in practice
9	absolutely more important	the evidence favouring one over the other is of the highest possible validity
2,4,6,8	intermediate values	when compromise is needed

### 3.10. Assessment of land and water potential for irrigation

The suitability of land for surface irrigation was determined from both groundwater and surface water using the weighted overlay of all factors. A suitability threshold (85%) was considered to identify the most suitable area for irrigation based on Worqlul *et al*. [19]. The suitability map was developed separately for both water sources in the Abbay basin. The suitable land at different threshold levels, starting from 80% with a 2% increment, was done to get insight into the suitable area for surface irrigation at various threshold levels.

The suitable land for irrigation from surface water was compared with small- to large-scale existing irrigation projects command areas for irrigation diversion in the basin. The command areas of ten irrigation projects—Dedissa, Beles, Fincha, Megech (Robit and Seraba pump), Ribb, Koga, Jemma, Gilgel Abbay and Anger Abbay—were used to validate the suitable land at 85% threshold from the surface water source. Similarly, the suitable land for surface irrigation from groundwater at an 85% threshold was validated using groundwater depth data in some selected portions of the basin.

In addition, the low flow potential of rivers in the basin for irrigation was computed and compared with the suitable land area at the 85% threshold for the North Gojam sub-basin as a case study. The North Gojam sub-basin was selected because of data availability and access to stream flow. A total of 15 river gauging stations were considered. The low flow volume (90 percentile flow) of rivers was considered to compute the potentially irrigable land from rivers [55]. Low flow volume was divided by the net irrigation requirement of crops dominantly grown in the Abbay basin (sorghum, wheat and maize) [19], using equation (3.3) to get the potentially irrigable land from rivers without the need for storage structure. Crop water requirement was done using the crop water assessment tool (i.e. CROPWAT 8). The crop coefficient of the dominant crops was taken from FAO [74].

3.3 NIR = 1.6  × Kc  × ETo – ER,

where NIR, Kc, ETo and ER are net irrigation requirement (mm), crop coefficient (dimensionless), reference evapotranspiration (mm) and effective rainfall (mm), respectively. The coefficient 1.6 behind Kc was introduced to account for the inefficacy of surface irrigation due to water application losses, conveyance losses, losses for land preparation and leaching [55]. Details of surface irrigation losses in the region can be referred from Belay *et al*. [75].

## 4. Results and discussion

### 4.1. Land suitability for surface irrigation based on individual factors

The consistency ratio (CR) of a pairwise matrix was checked and found to be 0.078, and 0.071 for surface and groundwater sources, respectively. According to Chen *et al.* [76], the result was found trustworthy (CR ≤ 0.2). According to Saaty [77], the judgement during pairwise comparison becomes untrustworthy if the CR is far from 0.1, which shows our results are within acceptable limits. The procedures and approaches used to calculate the CR can be referred from Mu & Pereyra-Rojas [78]. The result from the pairwise matrix (table 4) showed that proximity to the river network was the most important factor (26%) followed by the slope (20%) and SCI (18%). Rainfall deficit and land use were found to be the least important factors, accounting for 6% and 4% weight, respectively, for surface irrigation. Similarly, the most important factors for surface irrigation from groundwater sources were depth to groundwater (30%), slope (20%) and SCI (18%), respectively, (table 5); whereas, road and land use were found to be the least important factors accounting for 6% and 4%, respectively.

**Table 4.** Pair wise comparison matrix and weight from rivers [19,40,42]. Note: SCI, RD and UR are soil capability index, rainfall deficit and urban centre, respectively.

factors	river	slope	SCI	population	RD	UR	road	land use	weight
river	1	2	3	3	4	3	2	5	26
slope	1/2	1	3	2	2	4	3	3	20
SCI	1/3	1/3	1	3	5	4	3	4	18
population	1/3	1/2	1/3	1	2	1	2	3	10
RD	1/4	1/2	1/5	1/2	1	1/3	1/3	3	6
UR	1/3	1/4	1/4	1	3	1	1	3	9
road	1/2	1/3	1/3	1/2	3	1	1	2	8
land use	1/5	1/3	1/4	1/3	1/3	1/3	1/1	1	4

**Table 5.** Pair wise comparison matrix and weight from groundwater [19,20,54]. Note: GWD is to mean groundwater depth.

factors	GWD	slope	SCI	population	RD	UR	road	land use	weight
GWD	1	3	3	3	4	3	5	7	30
slope	1/3	1	3	3	4	3	3	3	20
SCI	1/3	1/3	1	3	5	4	3	4	18
population	1/3	1/3	1/3	1	2	1	2	3	9
RD	1/4	1/4	1/5	1/2	1	2	2	2	7
UR	1/3	1/3	1/4	1	1/2	1	½	3	7
road	1/5	1/3	1/3	1/2	1/2	2	1	2	6
land use	1/7	1/3	1/4	1/3	1/2	1/3	½	1	4

**Figure 7.**
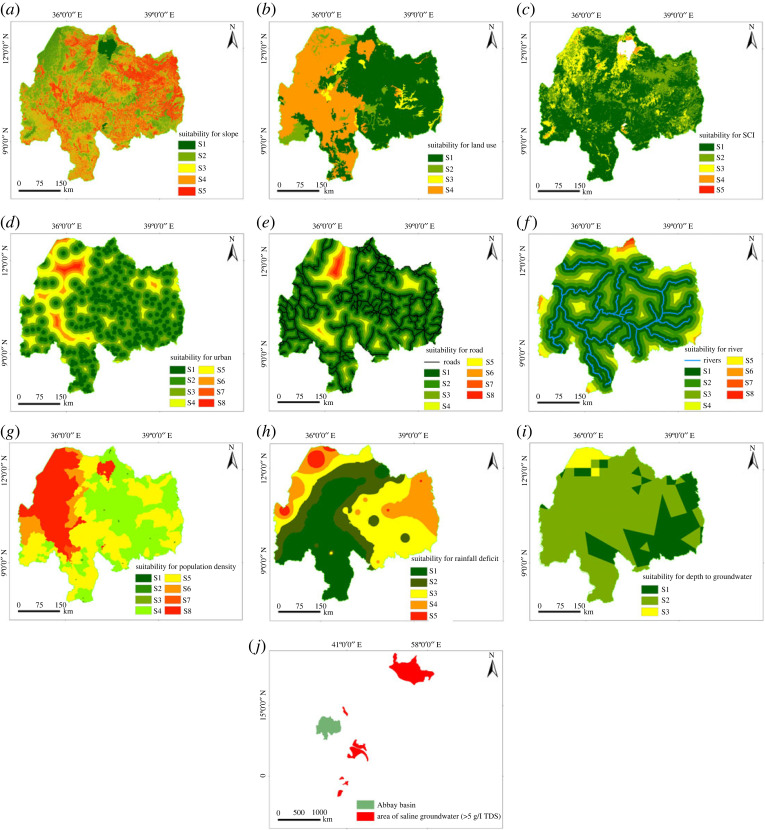
Land suitability for surface irrigation considering individual factors.

The suitability of land for surface irrigation based on individual criteria is presented in figure 7. Considering slope, about 6.3% of the Abbay basin, was found highly suitable (0–2%), 30% moderately suitable (2–8%); 13.3% marginally suitable (8–12%), 30.2% less suitable (12–30%) and 20.2% currently not suitable (greater than 30%), respectively, (figure 7*a*). The land-use suitability assessment showed that about 52% of the landmass (i.e. dominantly cultivated, moderately cultivated, irrigation, perennial crops and state farmland) was found highly suitable followed by 6.5% marginally suitable (i.e. shrub and bush land) (figure 7*b*). Similarly, suitability based on SCI showed that about 63% of the landmass was found highly suitable indicating good water-holding capacity of soil which is well drained (figure 7*k*) and deep soil depth (figure 7*j*). Moderately suitable land use which requires relatively less investment for land preparation was found at about 27% of the landmass (figure 7*c*).

Considering the proximity to the road network, urban centre and river network (figure 7*d–f*); the highly suitable landmass was found at 52%, 24% and 42%, respectively. On the other hand, the densely populated regions (i.e. 16 234–46 324 persons km^−2^) cover 15 km^2^ of the basin (figure 7*g*). About 36% of the basin has a sparsely populated area which had 364–809 persons km^−2^. According to the rainfall deficit suitability rating (figure 7*h*), about 50% of the basin area is suitable for surface irrigation, (i.e. highly suitable (31%) and 20% marginally suitable). Considering depth to groundwater (figure 7*i*), about 25% of the basin was highly suitable, and 71% was a moderately suitable area. The very shallow depth (0–7 m below ground level) is accessed in 25% of the basin area, and a large share (71% of the basin area) has been covered by a moderately suitable groundwater depth. The effect of salinity on irrigation (figure 7*l*) was evaluated based on the TDSs and found suitable for the entire basin. The concentration of TDS for the entire basin was below 5 g l^−1^ and is moderately suitable [70].

### 4.2. Land suitability assessment from rivers

**Figure 8.**
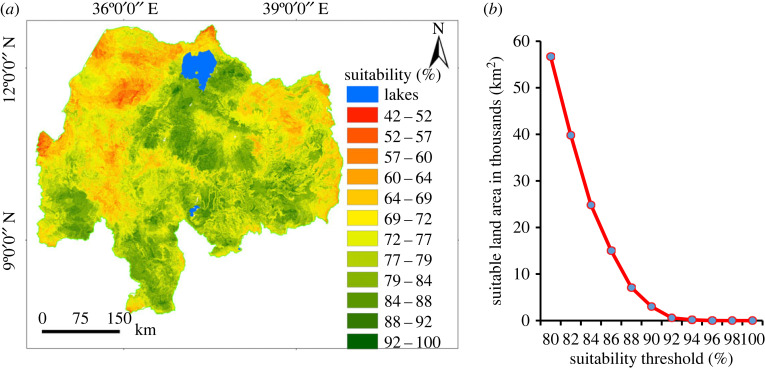
Land suitability for surface irrigation; suitability map (*a*) and suitability of land at different threshold levels (*b*).

**Figure 9.**
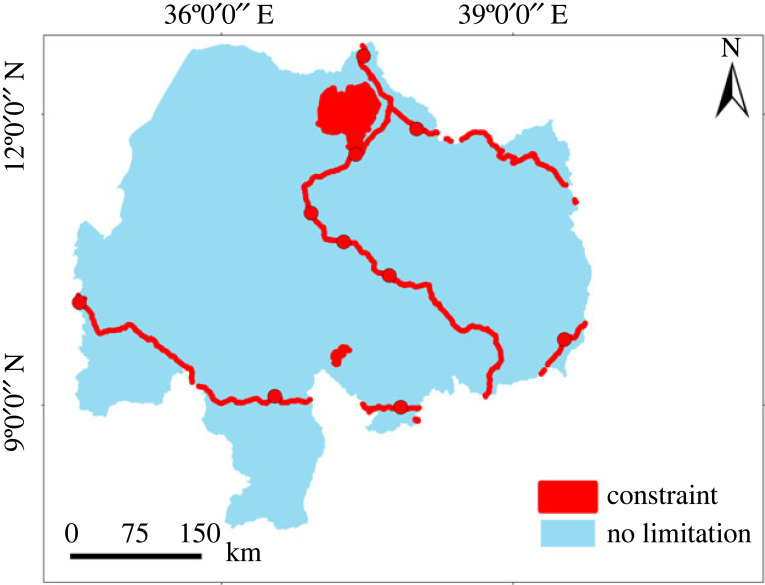
Constraint map.

The suitability map (figure 8*a*) combining all factors showed a value ranging from 42% (least suitable) to 100% (most suitable land). After multiplying with the constraint map (figure 9), the range of suitability ranges from 0% (least suitable) to 100% (highly suitable). The percentage of suitable land for surface irrigation from rivers was extracted by the 16 sub-basins of the Abbay basin at an 85% threshold level (table 6). The result showed that the highest suitable land for surface irrigation was found in the South Gojam sub-basin (23%), followed by Fincha (22.9%) and Tana (21.5%) sub-basins. The suitable land area at different threshold levels for the Abbay basin is presented in figure 8*b* with a 2% increment (from 80% to 100%) for the prioritization of irrigation investments.

**Table 6.** Land suitability for surface irrigation from rivers across Abbay sub-basins at 85% threshold level.

name of sub-basin	area of sub-basin (km^2^)	suitable area (ha)	percentage of suitable area
Anger	7878	72351.12	9.2
Beles	14 202	53823.58	3.8
Beshelo	13 184	8843.18	0.7
Dabus	20 241	200112.28	9.9
Didissa	19 229	242551.74	12.6
Dinder	14 807	250.52	0.09
Fincha	3923	89879.55	22.9
Guder	6937	101402.61	14.6
Jemma	15 628	169310.31	10.9
Muger	8129	132801.27	16.4
North Gojam	14 379	139621.51	9.7
Riad	8192	763.95	0.1
South Gojam	16 739	387977.50	23.2
Tana	11 975	256861.49	21.5
Welaka	6415	37768.69	5.9
Wenbera	12 952	22195.86	1.7

### 4.3. Land suitability for surface irrigation from shallow groundwater

**Figure 10.**
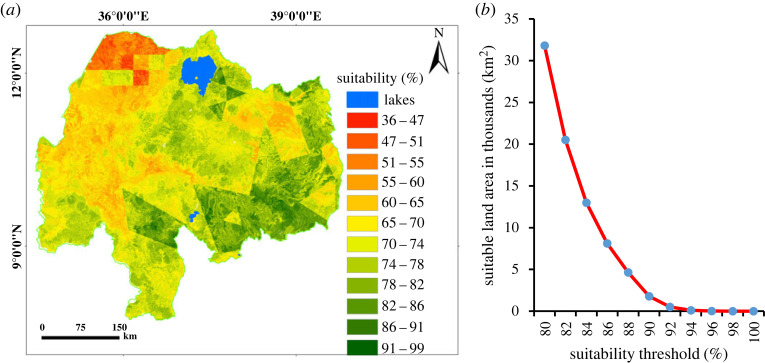
Land suitability for surface irrigation from shallow groundwater; land suitability (*a*) and suitability of land at different threshold levels (*b*).

The suitable land for surface irrigation from shallow groundwater ranges from 36% to 99% in order of suitability from least suitable to most suitable (figure 10*a*). The suitable land was extracted at an 85% threshold level for the 16 sub-basins and the potentially irrigable land is presented in table 7. The result showed Muger, Jemma, Guder, Anger and Fincha had 26%, 19%, 17%, 14% and 13% suitable land shares, respectively. Figure 10*b* depicts the irrigable area at the different threshold levels for the prioritization of irrigation investments.

**Table 7.** Potentially irrigable land from shallow groundwater across Abbay sub-basins at 85% threshold level.

name of sub-basin	area of sub-basin (km^2^)	suitable area (ha)	percentage of suitable area
Anger	7878	110937.74	14.1
Beles	14 202	565.69	0.04
Beshelo	13 184	73169.43	5.6
Dabus	20 241	3911.97	0.2
Didissa	19 229	32614.43	1.7
Dinder	14 807	0.10	0.0
Fincha	3923	50094.42	12.8
Guder	6937	117629.82	17.0
Jemma	15 628	300869.76	19.3
Muger	8129	210453.79	25.9
North Gojam	14 379	25839.58	1.8
Riad	8192	307.64	0.04
South Gojam	16 739	12482.79	0.7
Tana	11 975	41729.30	3.5
Welaka	6415	51289.31	8.0
Wenbera	12 952	4464.50	0.3

### 4.4. Low flow potential of rivers for irrigation

**Figure 11.**
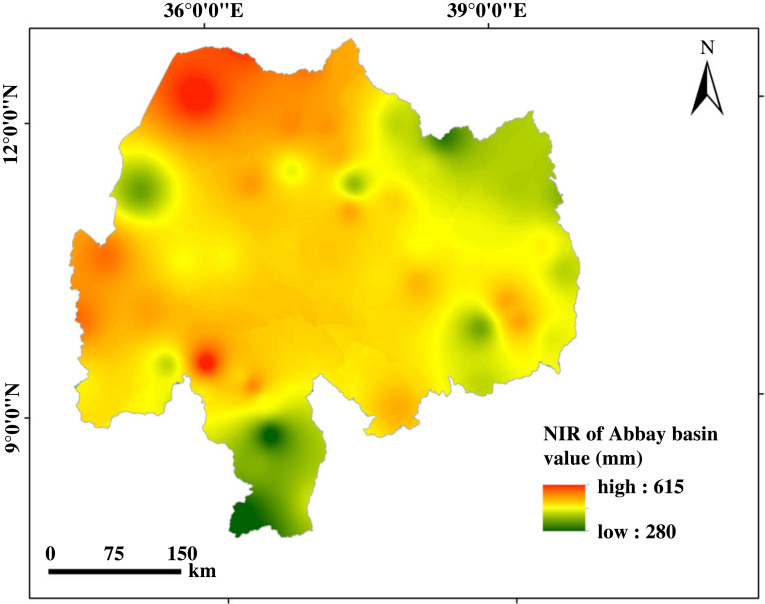
Net irrigation requirement. NIR (mm) for Abay basin.

The low flow potential of rivers (stream flow) for surface irrigation located in the North Gojam sub-basin is shown in table 8. The result showed a total of around 2263 km^2^ of potentially irrigable land from rivers in the North Gojam sub-basin. Among the rivers in the sub-basin, the Abbay river contributes the greatest low flow potential (1015.84 Mm^3^) for irrigation. The largest share of potentially irrigable land from the Abbay river was found at around 2199 km^2^ followed by the Andassa river (38 km^2^). Based on the low flow potential of rivers for irrigation, 64.5 km^2^ of irrigable land could be met through rivers except for the Abbay River. The result also showed around 97% of the potentially irrigable land from rivers of the North Gojam sub-basin was contributed by the Abbay River. The maximum and minimum net irrigation requirement for the entire Abbay basin was found to be 615 and 280 mm, respectively (figure 11). The range of net irrigation requirements in the North Gojam sub-basin was from 452 to 503 mm (table 8). In addition, the potential of all rivers for surface irrigation in the North Gojam sub-basin (2263 km^2^) was found greater than the potential suitable irrigable land area from the surface water source at 85% threshold level (1396.2 km^2^).

**Table 8.** Low flow potential of rivers to address suitable land for surface irrigation in the North Gojam sub-basin. NIR and Q are net irrigation requirement (mm) and stream flow volume (Mm^3^) at 90% exceedance probability.

river name	NIR (mm)	*Q* (@90%) (Mm^3^)	irrigation potential	
			km^2^	ha
Abbay	462	1015.84	2198.78	219877.71
Andassa	470	18.01	38.32	3831.91
Azuari	473	2.26	4.78	477.80
Chena	435	0.19	0.43	43.45
Ezana	485	0.22	0.44	44.43
Mendel	455	0.34	0.75	74.73
Muga	503	1.66	3.29	329.03
Sedie	482	1.46	3.02	301.87
Suha	481	1.11	2.30	229.73
Tigdar	468	0.65	1.38	137.82
Wenka	430	0.15	0.36	35.81
Shina	452	2.25	4.97	496.68
Shegez	452	0.73	1.62	161.50
Tull	450	0.39	0.87	87.22
Zemma	470	0.72	1.53	153.19

### 4.5. Validation of land suitability assessment

The comparison of suitable land for surface irrigation from rivers at an 85% threshold and the corresponding existing irrigation projects for the selected basin is shown in table 9. The percentage area of intersection for irrigation projects of Fincha, Ribb, Koga and Gilgel Abbay was found 100%. Similarly, Anger, Tiss Abbay and Jemma irrigation projects have a large percentage of intersections; 94%, 92% and 88%, respectively. The last two irrigation projects (i.e. Beles and Dedissa) share the least percentage area of intersection; 40% and 30%, respectively. On average, about 83% area of the intersection was found from the existing irrigation projects as compared with the 85% suitable land assessment.

**Table 9.** Comparison of suitable land area at 85% threshold with the existing irrigation projects (ground truth).

name of irrigation project	irrigation potential (ha)	suitable land at 85% threshold (ha)	percentage area of intersection
Fincha	20 000	19 715	100
Ribb	14 600	17 997	100
Koga	7004	9599	100
Gilgel Abbay	9980	13 422	100
Anger	14 450	13 528	94
Tiss abbay	14 928	13 797	92
Jemma	11 615	10 276	88
Beles	75 000	30 148	40
Dedissa	80 000	24 175	30

**Figure 12.**
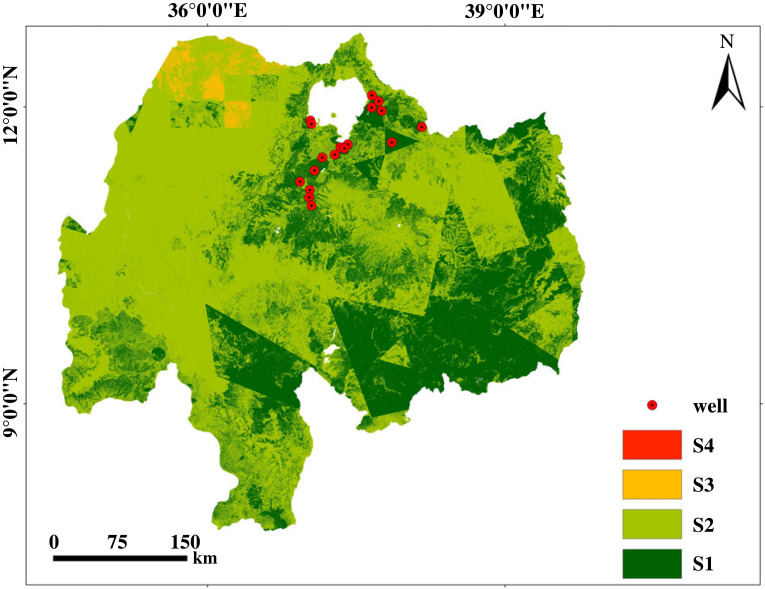
Validation of suitable land from shallow groundwater using depth to wells. The red-coloured points on the map are those wells selected for validation having a range of groundwater depth from 0 to 29 m.

Similarly, the suitable land from shallow groundwater computed with an 85% threshold was compared with existing well depths (figure 12). A total of 18 shallow groundwater wells having a maximum well depth of 29 m below ground level were selected for validation. The result showed that more than 73% of wells used for validation were located on suitable land for surface irrigation from groundwater sources.

## 5. Discussion

Land suitability assessment done in different parts of Ethiopian highlands indicated a promising result for surface irrigation practice [19,20,38,39,42,44]. In the Shaya river sub-basin in Oromiya, around 47% of the land was found suitable for surface irrigation from surface water sources [22]. The suitability analysis from individual factors according to the study indicated soil, land use land cover and slope were dominating, accounting for 98%, 93% and 66% of suitability, respectively. Similar work done on the Gilo sub-basin of Gambella [39] reported around 9% of the land is suitable for surface irrigation. The individual factors suitability from this study indicated around 92%, 90% and 87% suitability from land use/land cover, soil and slope, respectively. Results from our findings indicated that SCI, land use/land cover and slope had suitability of land for surface irrigation 99%, 61% and 50% of landmass, respectively. The slope of land has a greater impact on surface irrigation for gravity flow, and its impact on suitability elaborated according to Worqlul *et al*. [54] indicated it has an impact on land preparation, which affects a significant initial investment that weakens the economic feasibility.

Studies on the Lake Tana basin by Worqlul *et al*. [42] showed that 130 000 ha (11%) of land is suitable for surface irrigation from surface water sources, which is slightly lower than our findings (21.5%). A similar estimate was also reported by Assefa *et al*. [20] on the Tana basin, which is 21% considering water storage structures. Similarly, [38,41] reported that 68% and 93% of the landmass in the Dirma watershed and the Rib and Gumara watershed located in the upper Blue Nile was found suitable for surface irrigation from rivers, respectively. Worqlul *et al*. [19] reported groundwater-surface irrigation potential for Ethiopia to be around 8%. The study also showed Abbay basin is mostly dominated by shallow groundwater wells (less than 25 m) with 5706 mm groundwater storage, 3.8 l s^−1^ aquifer yield and approximately 21 186 km^2^ (11%) of suitable land for irrigation at an 85% suitability threshold. The potential of irrigable land in the Abbay basin using surface water and groundwater sources was found at 10% (19 165 km^2^ of land) and 5% (10 364 km^2^ of land), respectively, according to our study. The combined potential of groundwater and surface water was found to be 15% (29 529 km^2^ of land). This indicated a slight reduction of suitable irrigable land from groundwater to the previous study. This might be due to the coarse spatial resolution used for suitability factors and the method of evapotranspiration estimation used and adopted by the previous study. In addition, a little study on land suitability assessment from surface water in the Abbay basin was reported [44,46]. According to Yalew *et al*. [44] around 57 050 km^2^ (28.6%) highly suitable, 97 812 km^2^ (48.9%) moderately suitable land for irrigation was found which is far beyond our findings. This is because of two reasons: (i) suitability factors used in the study were somewhat different and (ii) reclassification of the factors for surface irrigation suitability was not based on FAO standards. Similarly, Yimer & Assefa [46] reported around 738 138 ha of suitable land for surface irrigation from rivers and irrigation projects using the Mike Hydro model, but the analysis was full of assumptions and scenarios based on 50% irrigation efficiency considering different cropping patterns.

Irrigation requirement indirectly determines the size of irrigable land and needs careful investigation. The range of net irrigation requirements for Ethiopian highlands ranged from 830 to 1630 mm [19] but our findings showed a range of 280–618 mm for the Abbay basin. The minimum net irrigation requirement across the nation (830 mm) was not even balanced by the maximum net irrigation requirement across the Abbay basin. This might be due to the adoption of MODIS global large spatial resolution evapotranspiration data to estimate the reference evapotranspiration and the choice of crops to estimate irrigation requirements by the former study.

**Figure 13.**
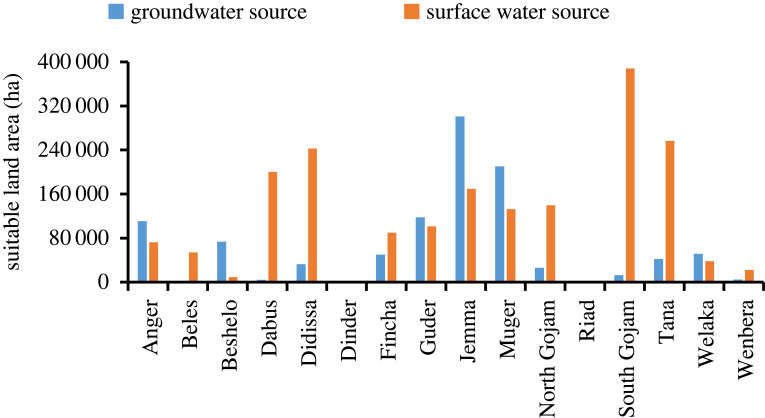
Comparison of suitable land area for surface irrigation from rivers and shallow groundwater sources at 85% threshold level for the Abbay sub-basins.

The suitable land for surface irrigation from shallow groundwater and rivers in each sub-basin is shown in figure 13. The result showed that about 23.2% (3879.78 km^2^) of the South Gojam sub-basin is suitable for surface irrigation from rivers, which was the highest among Abbay sub-basins, followed by Fincha (22.9%) and Tana (21.5%) sub-basins. Whereas, the Dinder sub-basin has the least suitable area from rivers (about 251 km^2^) mainly due to its higher elevation region dominated by steep slopes mostly greater than 30%. A previous study by Assefa *et al*. [20] on Lake Tana showed 21% suitable land for irrigation from surface water, considering water storage structures, which supports our findings. Similarly, the maximum suitable land percentage for surface irrigation from shallow groundwater was found at the Muger sub-basin (25.9% or 2104.54 km^2^) followed by Jemma (19.3%) and Anger (14%). This is due to the suitability of slope, land use/land cover type, SCI and depth to groundwater for irrigation compared with others in the basin for surface irrigation. The most suitable sub-basins for surface irrigation using surface water and groundwater (figure 13) were, Anger (9%,14%), Fincha (23%,19%), Guder (15%,17%), Jemma (11%,19%) and Muger (16%,26%) sub-basins, respectively. This is because these areas have suitable land for surface irrigation in terms of slope, SCI, population and proximity to urban and roads, respectively. Dinder, Riad and Wenbera sub-basins were the least suitable sub-basins for irrigation. The percentage area of intersection between irrigation potential of existing irrigation projects and suitable land assessment at 85% threshold at selected basins from Abbay basin showed 40% and 30% for Beles and Dedissa, respectively. The reason might be that the designer might not consider the different land suitability factors for surface irrigation, because most of the time designers may favour slope for gravity flow to happen to meet the farthest command area over the other factors.

## 6. Conclusion

This research was done to evaluate the potential of irrigable land and water availability, considering both surface water and groundwater sources using GIS-MCE techniques in the Abbay basin. A total of nine factors were selected to identify the suitable land for surface irrigation. Groundwater depth availability, proximity to the river and the slope were found the dominant factors affecting the practice of surface irrigation, and their corresponding weights were 30%, 26% and 20%, respectively. The potentially irrigable land from surface and groundwater sources at 85% suitability threshold was, respectively, 10% and 5.3% of the entire Abbay basin. The suitable land assessment was validated and found reasonable. Existing irrigation projects in the Abbay basin and their irrigation potential were used to validate the surface water source irrigation suitability. The result indicated around 83% of the area of intersection from the entire Abbay basin. Similarly, 73% of the area of the intersection was found from the entire Abbay basin for groundwater source irrigation validated using the existing well depths in the basin.

The potential of stream flow for surface irrigation was assessed based on the crop water requirement of dominating crops cultivated in the region (sorghum). The maximum and minimum irrigation requirement across the Abbay basin was found to be 615 and 280 mm, respectively. The result showed almost 97% of the potentially irrigable land from stream flow was contributed by the Abbay River. A total of around 2263 km^2^ of land could be irrigated from stream flow in the North Gojam sub-basin. The potential of rivers except Abbay contributed only 64.5 km^2^ of irrigable land area in the sub-basin. The most suitable sub-basins for surface irrigation using surface water and groundwater were Anger (9%,14%), Fincha (23%,19%), Guder (15%,17%), Jemma (11%,19%) and Muger (16%,26%) sub-basins, respectively. Dinder, Riad and Wenbera sub-basins were the least suitable sub-basins for surface irrigation. The results from this study would help local decision-makers and stakeholders with the expansion of small- to large-scale irrigation projects in suitable regions. In addition, it will give insight to agricultural experts on where to supply agricultural inputs and advisory services to farmers to increase agricultural productivity in the basin.

## Data Availability

The data can be found at Dryad https://doi.org/10.5061/dryad.zgmsbccds [79].
